# Clinical efficacy of Rifampicin and Streptomycin in combination against *Mycobacterium ulcerans* infection: a systematic review

**DOI:** 10.11604/pamj.2013.15.155.2341

**Published:** 2013-08-29

**Authors:** Marius Zambou Vouking, Violette Claire Tamo, Carine Nouboudem Tadenfok

**Affiliations:** 1 Center for the Development Best Practices in Health, Yaoundé Central Hospital, Henri-Dunant Avenue, Messa, Yaoundé, Cameroon; 2 Catholic University for Central Africa, School of Health Sciences, Yaoundé, Cameroon

**Keywords:** Clinical efficacy, Mycobacterium ulcerans infection, Antibiotics treatment, Rifampicin, Streptomycin

## Abstract

Buruli ulcer (BU) is a cutaneous neglected tropical disease caused by Mycobacterium ulcerans. Synthesizing the evidence on their efficacy of antibiotic in the management of BU can help to better define their roles, identify weaknesses and inform clinicians on relevant measures than can be used to control BU. Our objectives is to assess the clinical efficacy of Rifampicin-Streptomycin given for 8 weeks of treatment of early M. ulcerans infection. We searched the following electronic databases from January 2005 to July 2012: Medline, EMBASE (Excerpta Medica Database), The Cochrane Library, Google Scholar, CINAHL (Cumulative Index to Nursing and Allied Health Literature), WHOLIS (World Health Organization Library Database), LILACS (Latin American and Caribbean Literature on Health Sciences) and contacted experts in the field. There were no restrictions to language or publication status. All study designs that could provide the information we sought for were eligible provided the studies were conducted in the third world. Critical appraisal of all identified citations was done independently by three authors to establish the possible relevance of the articles for inclusion in the review. Of the 115 studies, 09 papers met the inclusion criteria. The duration of treatment ranged from 8 to 48 weeks depending on the severity. Oral chemotherapy alone obtained a curative rate of 50%. The “dual” mode of treatment (surgery + chemotherapy) reduced hospital admission period from 90 to 39.8 days, that's to 44.2%. This treatment for early stages could therefore replace surgery and in severe cases, is an indispensable aid before surgery. These results confirmed that the daily administration of Rifampicin and Streptomycin is an effective treatment for M. ulcerans infection in an early stage. Subsequent systematic reviews should be conducted to determine if antibiotics could heal injuries without resorting to surgery and to compare different treatment durations.

## Introduction

Buruli ulcer (BU) is a cutaneous Neglected Tropical Disease (NTD) caused by *Mycobacterium ulcerans* [1–3]. It is the third most frequent mycobacterial infection after tuberculosis and leprosy [1, 2]. A plasmid of *M. ulcerans* encodes the production of mycolactone [3], an immunomodulatory Macrolide toxin that causes tissue necrosis [4].

Many antimycobacterial agents show activity against *M. ulcerans* in vitro, and experiments in animals, such as the mouse footpad model, show that Streptomycin in combination with Rifampicin is highly bactericidal [5, 6]. In a pilot study sponsored by WHO, 31 patients clinically diagnosed with pre-ulcerative *M. ulcerans* infection were treated with Streptomycin and Rifampicin for 0, 2, 4, 8, or 12 weeks [7]. In 2000, the WHO Advisory Group on Buruli ulcer recommended a study to examine the possible benefit of antibiotic treatment in human subjects [7]. With very convincing results obtained in Ghana and Benin the WHO has published a guide on the establishing antibiotics in the treatment of BU.

On the basis of these findings, preliminary guidelines were issued by the WHO recommending Streptomycin together with Rifampicin as a standard treatment for *M. ulcerans* infection, [8] with or without additional surgical debridement or skin grafting. Synthesizing evidences on the efficacy of antibiotic in the management of BU can help to better define their roles, identify weaknesses and inform clinicians on relevant measures than can be used to control BU. We therefore conducted a systematic review to summarize the evidence of the clinical efficacy of Rifampicin and Streptomycin on the control of BU.

### Objectives

The aim of this study is to assess the clinical efficacy of Rifampicin-Streptomycin given for 8 weeks for the treatment of early *M. ulcerans* infection. The specific objectives of this study are to: rReview the current state of knowledge on the activities of Rifampicin and Streptomycin combined against *M. ulcerans* infection; to determine which oral antibiotics is more effective for managing BU with or without surgical excision.

## Methods

### Search strategy

We searched the following electronic databases from January 2005 to July 2012: Medline, EMBASE (Excerpta Medica Database), the Cochrane Library, Google Scholar, CINAHL (Cumulative Index to Nursing and Allied Health Literature), WHOLIS (World Health Organization Library Database), LILACS (Latin American and Caribbean Literature on Health Sciences) and contacted experts in the field. There were no restrictions to language or publication status. Our search was limited to the last seven years, as they correspond to the period of where the WHO validated antibiotic treatment of BU [8]. We used the following terms in our search: *clinical efficacy, Rifampicin and Streptomycin, antibiotic treatment, Buruli ulcer, Mycobacterium ulcerans, and outcome*.

**Study design:** All study designs were eligible for inclusion provided they were on antibiotic treatment.

**Study participants:** Owing to the wide range of functions that fall under the umbrella term “clinical efficacy” we designed a definition of our own. For this review, we defined antibiotic treatment as individuals trained in the particular role of delivering curative or preventive care in the management of BU.

**Types of interventions:** We included interventions if the description was adequate for us to establish that it was an antibiotic treatment of BU. Where such details were unclear, we contacted the study authors, whenever possible, for more information.

**Outcomes:** Any of the following outcomes were sought:

1. Type of treatment
2. Clinical efficacy of Rifampicin and Streptomycin
3. Side effects
4. The role of the antibiotic treatment in the efficacy of surgery

**Data extraction and management:** Critical appraisal of all identified citations was done independently by two authors (VCT and CNT) to establish the possible relevance of the articles for inclusion in the review. Studies were reviewed for relevance based on types of participants, interventions (management of BU), and outcome measures. We retrieved full text copies of the articles identified as potentially relevant by either one or both review authors. Where appropriate, we contacted study authors for further information and clarification. Disagreements were resolved by consensus or by arbitration of a third review author (MZV). Data are reported in a narrative manner.

**Assessment of quality in included studies:** The included studies were not scored for quality.

## Current status of knowledge

**Figure 1:**
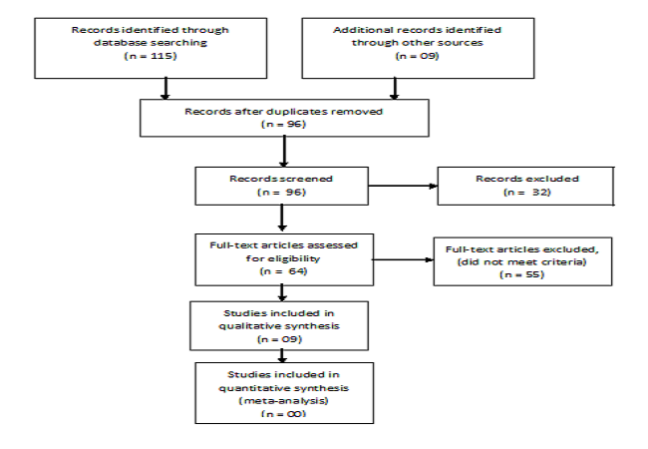
PRISMA flow diagram

Our searches retrieved 115 studies, of which 09 are included in the review (Figure 1, Table 1).

**Table 1:** Profile of different studies on the Buruli ulcer treatment in the world

	Reference	Type of study	Country	Type of antibiotic association	Number of weeks of treatment	Route of administration	Number of patients
**1**	Chauty et al., 2007 [20]	Cohort study	Benin	Streptomycin-Rifampin	8 weeks	Injection	224
**2**	Chauty et al., 2011 [13]	Cohort study	Benin	Streptomycin-Rifampin	8 weeks	Per os	30
**3**	Etuaful et al., 2005 [7]	RCT	Ghana	Streptomycin-Rifampin	12 weeks and surgery was performed after 4 weeks	Injection and per os	Not reported
**4**	Gordon et al., 2010 [10]	RCT	Autralia	Streptomycin-Rifampin	between 4 and 8 weeks	Per os	04
**5**	Kibadi et al, 2010 [14]	Cohort study	DRC	Streptomycin-Rifampin	12 weeks and surgery was performed after 4 weeks	Injection	92
**6**	O'Brien et al., 2012 [11]	Cohort study	Australia	Streptomicin-Rifampicin	8 weeks	Per os	133
**7**	Nienhuis et al., 2010 [9]	RCT	Ghana	Streptomicin-Rifampicin	8 weeks	Injection et per os	151
**8**	Saka et al., 2012 [12]	Cohort study	Togo	Streptomicin-Rifampicine	8 weeks	Injection and per os	119
**9**	Agbenorku et al., 2007 [16]	Cohort study	Ghana	Streptomicin-Rifampicin	8 weeks	Injection et per os	62

### Study characteristics

Nine studies including 3 Randomised Controlled Trials (RCTs) [7, 9, 10] and 06 cohorts [11–14, 16, 20] met the inclusion criteria (Figure 1).

**The different combinations and mode of administration of antibiotics:** All nine studies evaluated the efficacy of combinationing Streptomycin-Rifampin in confirmed BU patients [7, 9–14, 16, 20]. Three studies have evaluated the oral treatment [10, 11, 13], 02 studies treatment injection [14–20] and 02 studies compared treatment with oral and one by injections of antibiotics [7–9].

**Antibiotic treatment associated with surgery:** The surgery was performed after an antibiotic treatment in three studies [10, 12, 13]. Lesion size at baseline was the main factor associated with surgery. The duration of treatment ranged from 8 to 48 weeks depending on the severity and the mean duration of treatment was 8 weeks according to the WHO recommendations [9, 11–13, 16, 20]. The average recovery period was 104 days (range, 30-212 days) [13].

**Clinical efficacy of Rifampin-Streptomycin:** Oral chemotherapy alone obtained a curative rate of 50% [13]. One participant developed an injection abscess and two others developed an abscess near the lesion, which was incised and drained [9]. Saka and colleagues reported complications in 20 patients of which three were Amputated [12].

Regarding the Polymerase Chain reaction (PCR) results after treatment Chauty and collaborators reported negative PCR results after treatment in 14 of the 27 samples analyzed, Etuaful and colleagues reported 07of the 21 samples negative by PCR, histopathology [7], suggesting the persistence of mycobacterial material, as described by others.

The “dual” mode of treatment (surgery + chemotherapy) reduced hospital admission period from 90 to 40 days, that's to 44.2%. This would directly reduce the cost of treatment for the BU patients [15].

The WHO has recommended the use of Rifampicin-Streptomycin (15 mg/kg once daily intramuscular streptomycin and 10 mg/kg Rifampin orally once daily) for the treatment of*Mycobacterium ulcerans* infection for a period of 8 weeks for first category lesions. Surgery comes in depending on the size of the lesion [8].

Several authors have attempted to evaluate the effectiveness of different antibiotic therapies in the treatment of BU [7, 9, 10, 12, 13, 16] and most studies have evaluated the clinical efficacy of two different regimes of combination therapies [7, 9–13, 16, 20].

After haven completed the antibiotic therapy, patients should be followed for at least 10 months (a total of 12 months from the start of treatment) to confirm healing, evaluate eventual complications and notice any relapses [8].

All these results were obtained without toxic effect of antibiotics. The authors reported low treatment failure (below 40%) relative to the association Rifampin-Streptomycin [9, 10, 12, 13] and less than 20% Rifampin-Streptomycin associated with surgery [7, 12, 13].

Chemotherapy by injection only managed to get a 47% cure and was particularly effective against ulcers less than 5 cm in diameter and no side effects were reported [20]. Oral chemotherapy alone obtained a curative rate of 50% [13].

This treatment happens to be of great help for the surgery because it sterilizes wounds, limits the size of the excisions and reduces the number of relapses. It turned out that the surgery was not required for early stages [11–13, 15, 16].

This treatment for early stages could therefore replace surgery and in severe cases, is an indispensable aid before surgery. Capable of being established at local health centres, this treatment could significantly reduce the cost of care and marginalization of patients. One participant developed an injection abscess and two others developed an abscess near the lesion which was incised and drained [9]. Saka and colleagues reported complications in 20 patients of which three were Amputated [12]. The average recovery period was 104 days (range, 30-212 days) [13].

Regarding the PCR results after treatment Chauty and collaborators reported negative PCR results after treatment in 14 of the 27 samples analyzed [13], Etuaful and colleagues reported 07 of the 21 samples negative by PCR, histopathology [7], suggesting the persistence of mycobacterial material, as described by others.

*M. ulcerans* culture test were positive at the initial stage that is to say before the treatment and two weeks after treatment. They were then negative, indicating that this antibiotic combination may be useful to support the nodules and closets, the only type of damage covered by this study. Preliminary analysis of the test results sponsored by WHO (Study I) showed that treatment with Rifampicin and Streptomycin managed to make negative cultures in 4 to 12 weeks, but not after 2 weeks. During the period of observation before excision, most lesions decreased in size and we have reasons to believe that the clinical treatment by antibiotics alone is effective. Currently, data from extensive research suggests that there is no significant difference between oral antibiotic therapy and parenterally in the treatment of BU [18, 19]. However, the available data support the hypothesis that oral treatment of both antibiotics may be used as effectively as parenteral therapy in this particular group of children [16, 17]. Ji and colleagues showed that regimens combining Rifampicin or Moxifloxacin were as effective as Rifampicin-Streptomycin in mice [18].

Studies by O'Brien, Alffenaar, Almeida employees and collaborators showed that Rifampicin, Amikacin and streptomycin had a bactericidal activity on M. ulcerans [11, 16, 23]. As Clarithromycin alone exhibited clear-cut bacteriostatic activity, an additive effect of the combination Rifapentine-Clarithromycin and even Rifampin-Clarithromycin was expected. Unfortunately the co-administration of a Rifamycin and Clarithromycin, both drugs given orally at doses equivalent to human doses was less effective than each Rifamycin alone in mice infected with *M. ulcerans* [23].

These results have important implications for the treatment of patients with BU in developing countries, including the reduction of pain associated with needle complications and patient discomfort during treatment.

## Conclusion

These results confirmed that the daily administration of Rifampicin and Streptomycin is an effective treatment for *M. ulcerans* infection at an early stage. Subsequent systematic reviews should be conducted to determine whether antibiotics could heal injuries without resorting to surgery and to compare different treatment durations.

## References

[1] WHO (2010). Global Experience of Community Health Workers for Delivery of Health Related Millennium Development Goals: A Systematic Review, Country Case Studies, and Recommendations for Integration into National Health Systems.

[2] Wansbrough-Jones M, Phillips R (2006). Buruli ulcer: emerging from obscurity. Lancet..

[3] Stienstra Y, van Roest MH, van Wezel MJ (2005). Factors associated with functional limitations and subsequent employment or schooling in Buruli ulcer patients. Trop Med Int Health..

[4] Van der Werf TS, Stinear T, Stienstra Y, van der Graaf WTA, Small PL (2003). Mycolactones and Mycobacterium ulcerans disease. Lancet..

[5] Havel A, Pattyn SR (1975). Activity of Rifampicin on Mycobacterium ulcerans. Ann. Soc. Belg. Med. Trop..

[6] Portaels F, Traore H, De Ridder K, Meyers WM (1998). In vitro susceptibility of Mycobacterium ulcerans to Clarithromycin. Antimicrob Agents Chemother..

[7] Etuaful S, Carbonnelle B, Grosset J (2005). Efficacy of the combination Rifampin-streptomycin in preventing growth of Mycobacterium ulcerans in early lesions of Buruli ulcer in humans. Antimicrob Agents Chemother..

[8] WHO (2005). Provisional guidance on the role of specific antibiotics in the management of Mycobacterium ulcerans disease (Buruli ulcer).

[9] Nienhuis WA, Stienstra Y, Thompson WA, Awuah PC, Abass KM, Tuah W, Awua-Boateng NY, Ampadu EO, Siegmund V, Schouten JP, Adjei O, Bretzel G, van der Werf TS (2010). Antimicrobial treatment for early, limited Mycobacterium ulcerans infection: a randomized controlled trial. Lancet..

[10] Gordon CL, Buntine JA, Hayman JA, Lavender CJ, Fyfe JAM (2010). All-Oral Antibiotic Treatment for Buruli Ulcer: A Report of Four Patients. PLoS Negl Trop Dis..

[11] O'Brien DP, McDonald A, Callan P, Robson M, Friedman ND (2012). Successful Outcomes with Oral Fluoroquinolones Combined with Rifampicin in the Treatment of Mycobacterium ulcerans: An Observational Cohort Study. PLoS Negl Trop Dis.

[12] Saka B, Landoh DE, Kobara B, Djadou KE, Yaya I, Yékplé KB, Piten E, Balaka A, Akakpo S, Kombaté K, Mouhari-Toure A, Kanassoua K, Pitché P (2013). Profile of Buruli ulcer treated at the National Reference Centre of Togo: a study of 119 cases. Bull Soc Pathol Exot..

[13] Chauty A, Ardant MF, Adeye A, Euverte H, Guédénon A, Johnson C, Almeida D, Converse PJ, Ahmad Z, Dooley KE, Nuermberger EL (2011). Activities of Rifampin, Rifapentine and Clarithromycin Alone and in Combination against Mycobacterium ulcerans Disease in Mice. PLoS Negl Trop Dis..

[14] Kibadi K, Boelaert M, Fraga AG, Kayinua M, Longatto-Filho A (2010). Response to Treatment in a Prospective Cohort of Patients with Large Ulcerated Lesions Suspected to Be Buruli Ulcer (Mycobacterium ulcerans Disease). PLoS Negl Trop Dis..

[15] Agbenorku P, Agbenorku M, Gotah E, Saundersonand P, Lehman L (2006). Benefits of a combination of surgery and chemotherapy in the management of Buruli the ulcer patients. J Sci Tech..

[16] Alffenaar JWC, Nienhuis WA, de Velde F, Zuur AT, Wessels AMA, Almeida D, Grosset J, Adjei O, Uges DRA, van der Werf TS (2010). Pharmacokinetics of Rifampin and Clarithromycin in Patients Treated for Mycobacterium ulcerans Infection. Antimicrobial Agents and Chemotherapy..

[17] Ji B, Chauffour A, Robert J, Lefrançois S, Jarlier V (2007). Orally administred combined regimens for treatment of Mycobacterium ulcerans infection in mice. Antimicrobial agents and chemotherapy..

[18] Ji B, Chauffour A, Aubry A, Robert J, Ibrahim I, Jarlier V (2009). Impacts of Dosing Frequency of the Combination Rifampin-Streptomycin on Its Bactericidal and Sterilizing Activities against Mycobacterium ulcerans in Mice. Antimicrobial agents and chemotherapy..

[19] Chauty A, Ardant MF, Adeye A (2007). Promising clinical efficacy of Streptomycin-Rifampin combination for treatment of Buruli ulcer (Mycobacterium ulcerans disease). Antimicrob Agents Chemother..

[20] Van der Werf TS, Stienstra Y, Johnson RC (2005). Mycobacterium ulcerans disease. Bull World Health Organ..

[21] Dega HA, Bentoucha J, Robert V, Jarlier, Grosset J (2002). Bactericidal activity of Rifampin-Amikacin against Mycobacterium ulcerans in mice. Antimicrob Agents Chemother.

[22] Buntine JC, Etuaful S, Johnson C, Grosset J, Portaels F, Wansbrough-Jones M (2004). Recommandations provisoires pour certains antibiotiques dans la prise en charge de l'infection à Mycobacterium ulcerans (ulcère de Buruli).

[23] Almeida D, Converse PJ, Ahmad Z, Dooley KE, Nuermberger EL (2011). Activities of Rifampin, Rifapentine and Clarithromycin Alone and in Combination against Mycobacterium ulcerans Disease in Mice. PLoS Negl Trop Dis..

